# Cell polarity and cell adhesion associated gene expression differences between invasive micropapillary and no special type breast carcinomas and their prognostic significance

**DOI:** 10.1038/s41598-021-97347-8

**Published:** 2021-09-16

**Authors:** Zsófia Kramer, István Kenessey, Ambrus Gángó, Gábor Lendvai, Janina Kulka, Anna-Mária Tőkés

**Affiliations:** 1 2nd Department of Pathology, Semmelweis University, Üllői Street 93, Budapest, 1091 Hungary; 2 1st Department of Pathology and Experimental Cancer Research, Semmelweis University, Üllői Street 26, Budapest, 1085 Hungary

**Keywords:** Breast cancer, Cancer genomics

## Abstract

Invasive micropapillary carcinoma of the breast (IMPC) has been in the focus of several studies given its specific histology and clinicopathological course. We analysed mRNA expression profiles and the prognostic value of 43 genes involved in cell polarity, cell-adhesion and epithelial–mesenchymal transition (EMT) in IMPC tumors and compared them to invasive breast carcinomas of no special type (IBC-NST). IMPCs (36 cases), IBC-NSTs (36 cases) and mixed IMPC-IBC NSTs (8 cases) were investigated. mRNA expression level of selected genes were analysed using the NanoString nCounter Analysis System. Distant metastases free survival (DMFS) intervals were determined. Statistical analysis was performed using Statistica 13.5 software. Twelve genes showed significantly different expression in the IMPC group. There was no difference in DMFS according to histological type (IBC-NST vs. IMPC). High CLDN3, PALS1 and low PAR6 expression levels in the entire cohort were associated with shorter DMFS, and PALS1 was proven to be grade independent prognostic factor. Positive lymph node status was associated with higher levels of AKT1 expression. Differences in gene expression in IMPC versus IBC-NST may contribute to the unique histological appearance of IMPCs. No marked differences were observed in DMFS of the two groups. Altered gene expression in the mTOR signaling pathway in both tumor subtypes highlights the potential benefit from AKT/mTOR inhibitors in IMPCs similarly to IBC-NSTs.

## Introduction

Invasive micropapillary carcinoma (IMPC) of the breast is a histologically well defined subtype of breast carcinoma, accounting for 1–8.4% of all breast carcinomas^1–3^. IMPC of the breast is characterized by tumor cells forming morules situated within empty stromal spaces and showing an inside-out staining pattern with epithelial membrane antigen (EMA). Several studies have shown that compared to IBC-NST, IMPC have a higher rate of locoregional recurrence, peritumoral lymphovascular invasion and axillary lymph node involvement^4,5^. In addition, according to some studies, long term survival proved to be better in IMPC^1,5,6^, while other studies have shown significantly worse outcomes in IMPC cases^7^. Recent studies have found no significant difference between the prognosis of the two subtypes^4,5,8–10^.

Despite the well known histological features of IMPC, the underlying mechanisms leading to its special appearance and the higher locoregional aggressiveness are not fully understood. Understanding the pathomechanisms leading to higher rate of locoregional recurrence and lymphovascular invasion may open new therapeutic opportunities for patients with IMPC. It is reasonable to consider that proteins playing role in cell-adhesion, cell polarity, cell junction and tight junction maintenance are involved in the special histological apparence of IMPCs.

Cell polarity protein complexes (such as Crumbs complex, Par complex, Scribble complex) are key factors in normal cellular and structural development as well as in epithelial apical-basal polarity and directed cell migration. Alterations of these complexes are very common in cancer formation and progression^11^. Gruel et al. found that cell polarity gene LIN7A plays a significant role in polarity defects associated with breast carcinomas, especially in IMPCs^12^.

Cell adhesion molecules and tight junction proteins are fundamental in tissue morphogenesis and signaling between cells and the surrounding extracellular matrix (ECM). These molecules have been studied in many cancer types, and their expression profile and localization pattern have been found to differ in normal and tumor tissue as well as in different types of carcinomas. The roles of these molecules in tumor progression and suppression have also been investigated by many groups^13,14^. The dysregulation of these molecules occurs during carcinogenesis via different mechanisms. One of this is the epithelial–mesenchymal transition (EMT), which is considered as a crucial step in tumor progression. The signaling pathways activated in these processes include WNT/B-CATENIN, JAK/STAT3 and PI3K/AKT pathways^14–16^.

The involvement of different chemokines and chemokin receptors in tumor progression is higly studied with fewer data outlining their role in the process of breast tumorigenesis and metastases in IMPC^17–20^. Among the studies focusing on the role of chemokines in breast cancer, some have shown that certain chemokines and their receptors play a role in the development of lymph node metastasis in breast cancer^20–22^.

In the everyday pathology practice, no unequivocal immunohistochemical markers, except for EMA—showing linear positivity at the periphery of the morule-like tumor cell clusters—are associated with IMPC. In this study we aim to reveal the eventual role of adhesion molecules, tight junction proteins, cell polarity and cancer signaling pathways genes as well as chemokine-chemokine receptors in IMPCs compared to IBC-NST cases and to analyze their prognostic values in the two groups.

## Materials and methods

**Figure 1:**
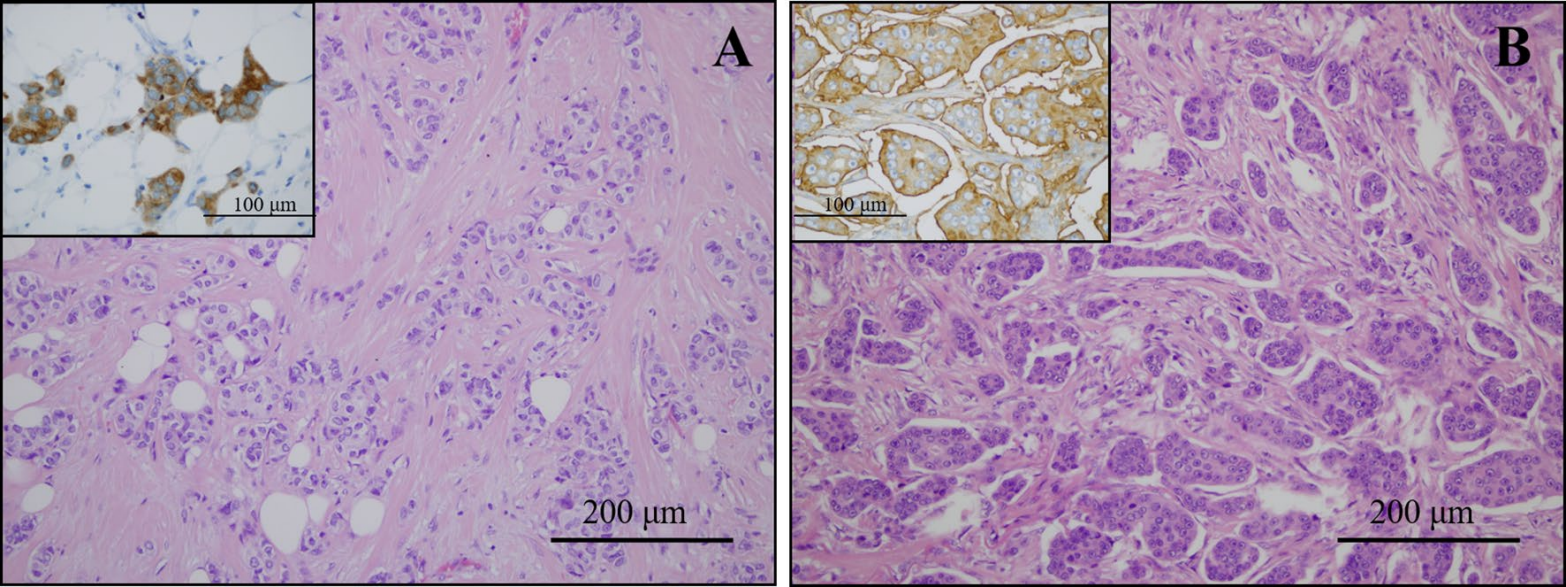
Histological characteristics of IBC-NST and IMPC tumors. Microscopic appearance of IBC-NST tumors (**A**) (H&E stain, ×20). Insert shows membranous/cytoplasmic positivity with EMA antibody (×60). Typical cluster formation in IMPC tumors (**B**) (H&E stain, ×20). Insert shows linear positivity at the periphery of the morule-like tumor cell clusters (inside-out pattern) with EMA antibody (×60).

During our study 36 cases of IMPC, 36 age- and stage-matched IBC-NST cases (for statistical comparison) and 8 mixed IMPC/IBC-NST cases were selected from the archive of the 2nd Department of Pathology (Semmelweis University Budapest) from the period of 2000 to 2018, with the ethical permission of Semmelweis University Research Ethics Committee (permission number: 240/2016). All cases were reviewed by expert pathologists and classified based on the World Health Organisation criteria^23^. After selecting the cases from our files mixed cases of micropapillary + tubular or mucinous carcinomas and the cases with missing clinicopathological- and follow-up data were excluded from this study. Additionally, IMPC cases were confirmed by the typical inside-out staining pattern of EMA immunohistochemistry (performed with automated Ventana BenchMark ULTRA system using Cell Marque Mouse Monoclonal antibody, 1:200)^24^ (Fig. 1). Tumor characteristics and patient data as well as clinical follow up information were obtained from the Semmelweis University Health Care Database and the National Cancer Registry.

### NanoString analysis

Using the NanoString nCounter Analysis System (NanoString Technologies, Seattle, WA), gene expression analysis was performed for each sample using a small, custom designed codeset. Genes, involved in cell-adhesion, tight junction, cell polarity and cancer signalling pathways including epithelial–mesenchymal transition in association with breast carcinomas were reviewed in the literature^11,12,15,17,22,25–31^. The selection of the genes for this study was performed based on the results of our previous research studies regarding the role of cell-adhesion- and tight junction molecules in breast cancer^32–35^, several other published results in this field and on cell polarity^11,12,16,29^ as well as considering the role of these genes as potential therapeutic targets or in chemoresistance according to the literature^36–38^. Additionally chemokine-chemokine receptor molecules mentioned in previous publications^17–22,39^ and considered to play a role in the development of lymph node metastasis, were also included (Supplementary Table 1 and Supplementary Table 2). Altogether 43 genes of interest and five housekeeping genes were selected.

### RNA isolation

Tumor cell content was defined prior to RNA isolation on hematoxylin–eosin (H&E) stained slides. In all analysed tumors, the proportion of tumor cells was more than 50%.

Three to five sections of 5 µm were cut from FFPE tissue blocks and placed in sterile Eppendorf tubes. In the cases of mixed IMPC/IBC-NST (8 cases altogether) the IMPC and IBC-NST component was separately macrodissected based on H&E stained slides and IMPC component was further analysed. After deparaffinisation, RNA was extracted from the tissue samples using the QIAGEN^®^ RNeasy^®^ FFPE Kit, according to the manufacturer’s instructions. RNA concentrations were measured with Quantus Fluorometer (Promega), and the samples were diluted to 30 ng/µl.

### NanoString nCounter mRNA analysis

mRNA hybridization was set up using the 12-tube PCR hybridization strips, Reporter CodeSet and Capture ProbeSet provided by NanoString. According to the manufacturers guide, 8 µl of Master Mix (Mixture of Reporter CodeSet and Hybridizaion Buffer) was added to 5 µl of sample mRNA (altogether 150 ng extracted mRNA) in a tube. After adding 2 µl of Capture ProbeSet to each tube, the solution was gently mixed, briefly spinned and placed immediately in a pre-heated 65 °C thermal cycler for 24–26 h. After incubation, the samples were immediately placed into the nCounter Prep station, and then analysed in the Digital Analyser (nCounter FLEX Analysis System). Measurements were taken at high sensitivity with 555 FOV.

### Statistical analysis

The raw expression data from the Digital Analyzer were normalized using nSolver version 4.0 (NanoString Technologies, Seattle USA). Briefly, the expression data were background corrected by using the geometric mean of the negative controls. The data then were normalised with the geometric mean of the five housekeeping genes. Median of expression values of examined genes was set as threshold. Expression values below median were defined as “low expression” and values above median as “high expression”.

Categorical data were compared using Chi-square or Fisher’s exact tests. Asymmetrical numeric data (IMPC vs. IBC-NST) were analyzed by Mann–Whitney test. Kaplan–Meier analysis was performed using DMFS as the endpoint. DMFS intervals were determined as the time period from initial diagnosis to the time of diagnosing distant organ metastasis. The comparison of survival functions for different strata was assessed with the log-rank statistics. Multivariate analysis of prognostic factors was performed using Cox's regression model.

Statistical significance was confirmed when p-values were < 0.05. Statistical analysis was performed using Statistica 13.5 software (TIBCO Software Inc, Palo Alto, CA).

To compare our results of the prognostic impact of selected genes (based on DMFS) with a large database the KM Plotter Online Tool, a publicly available database was selected^40,41^.

### Ethics approval and consent to participate

Ethics approval was provided by Semmelweis University Research Ethics Committee. Permission number: 240/2016.

Our retrospective study was carried out in accordance with the Helsinki Declaration.

In accordance with the Hungarian Research regulations, in case of retrospective studies, where patients are no longer trackable, possession of an Ethical approval provides excemption of the need of individual patient consent acquirement. Informed consent was waived by the Semmelweis University Research Ethics Committee.

All methods were carried out in accordance with relevant guidelines and regulations.

## Results

### Patients characteristics

Tumors of 80 breast cancer patients were included in our study. Mean age of the patients was 62 years in the IBC-NST group, 63 years in the IMPC and 65.5 in the mixed IMPC/IBC-NST group. Most of the patients presented with lymph node metastasis, and most of the tumors were grade 2, stage pT1-2. Median follow up time was 48 months (range: 0–230 months). Distant metastasis occured in 25 out of 80 cases (12/36 in IBC-NST, 11/36 in IMPC and 2/8 in mixed IMPC/IBC-NST cases).

Patients’ and tumors’ characteristics are shown in Table 1. All three patient groups showed similar distribution regarding age and prognostic factors.

**Table 1:** Patients’ and tumors’ characteristics.

	IBC-NST	IMPC	Mixed IMPC/IBC-NST	p-value
Total patient number	36	36	8	
Median years of age (range)	62 (37–83)	63 (33–85)	65.5 (34–69)	0.992^1^
Median of Ki67 LI (range)	15 (1–100)	15 (1–90)	23 (5–90)	0.579^1^
**Grade**				0.728^2^
I	3 (8.3%)	3 (8.3%)	1 (12.5%)	
II	20 (55.6%)	23 (63.9%)	3 (37.5%)	
III	13 (36.1%)	10 (27.8%)	4 (50%)	
**T**				0.702^2^
1	14 (38.9%)	14 (38.9%)	3 (37.5%)	
2	11 (30.6%)	8 (22.2%)	4 (50%)	
3	8 (22.2%)	9 (25%)	0 (0%)	
4	3 (8.3%)	3 (8.3%)	1 (12.5%)	
NA		2 (5.6%)		
**N**				0.94^2^
0	13 (36.1%)	11 (30.6%)	3 (37.5%)	
1	9 (25%)	9 (25%)	3 (37.5%)	
2	6 (16.7%)	3 (8.3%)	1 (12.5%)	
3	5 (13.9%)	7 (19.4%)	1 (12.5%)	
NA	3 (8.3%)	6 (16.7%)		
**ER**				0.121^2^
+	28 (77.8%)	34 (94.4%)	7 (87.5%)	
−	8 (22.2%)	2 (5.6%)	1 (12.5%)	
**PR**				0.018^2^
+	18 (50%)	28 (77.8%)	7 (87.5%)	
−	18 (50%)	8 (22.2%)	1 (12.5%)	
**HER2**				0.601^2^
+	5 (13.9%)	9 (20.4%)	1 (12.5%)	
−	31 (86.1%)	35 (79.6%)	7 (87.5%)	

*LI* labeling index (%).

^1^Kruskal-Wallis, ^2^Chi-square.

### Difference of gene expression pattern compared between IMPC and IBC-NST groups

**Figure 2:**
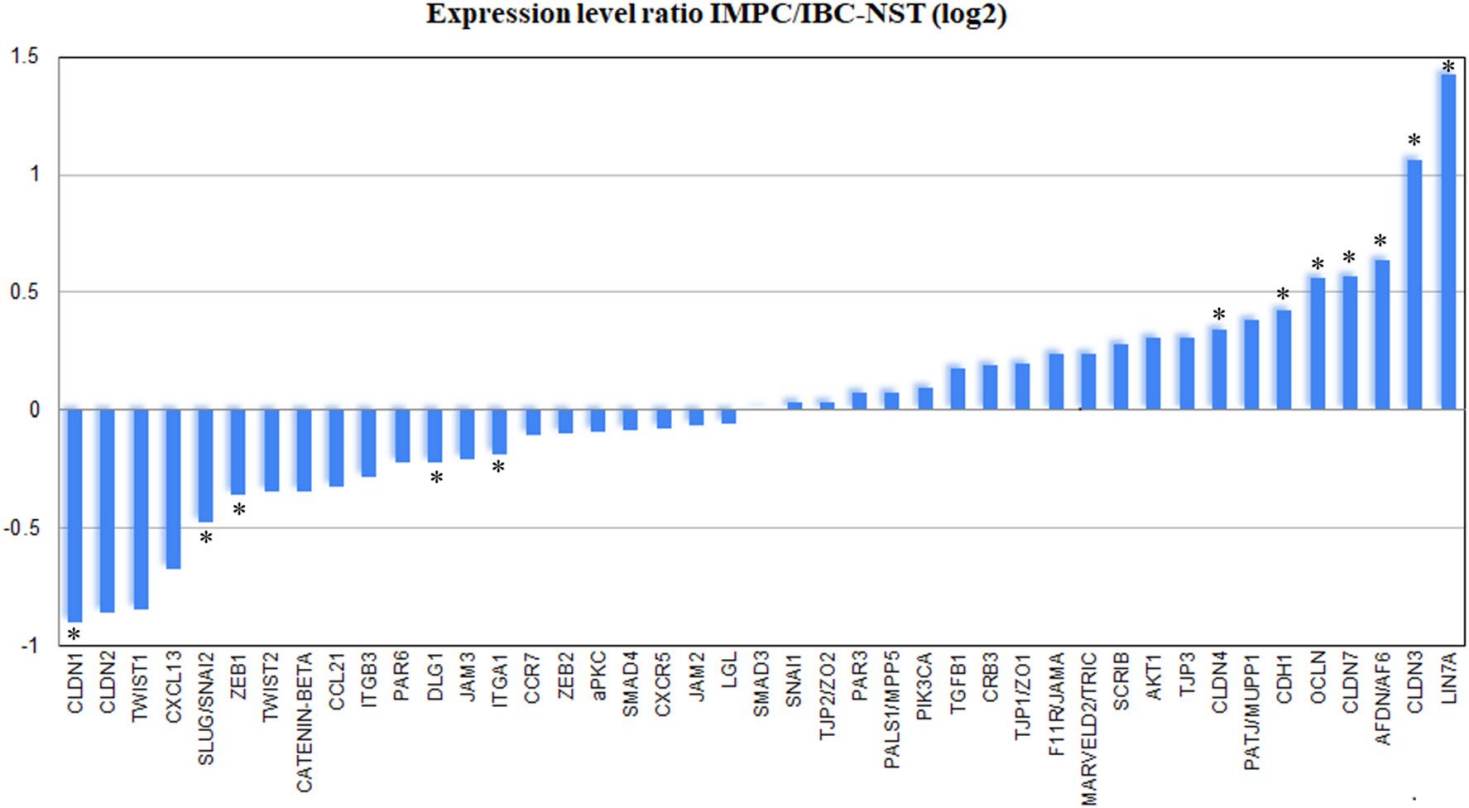
Gene expression ratio in IMPC/IBC-NST tumors. Diagram showing the gene expression ratio of IMPC/IBC-NST tumors in the examined 43 genes. X axis: 43 examined genes, Y axis: logarithm of ratio of IMPC/IBC-NST mRNA expression values. Asterix (*) marks genes showing significantly different expression values between the two tumor groups.

Since patient characteristics’ distribution was similar in IMPC and mixed groups, IMPC component of mixed tumors was included to the IMPC group for gene expression pattern comparison of IMPC and IBC-NST groups. Significant differences in the mRNA expression levels of 12 genes out of the examined 43 genes was detected (Supplementary Table 3 and respective heatmap shown on Supplementary Figure 1). In IMPCs the expression levels of AF6 (p = 0.000005), CLDN3 (p = 0.000005), CLDN4 (p = 0.002431), CLDN7 (0.000131), LIN7A (0.000081), CDH1 (0.01176), OCLN (0.000233) were significantly higher, while those of CLDN1 (p = 0.004673), DLG1 (0.002207), ITGA1 (0.044779), SLUG/SNAI2 (0.007495), ZEB1 (0.049064) were significantly lower (Fig. 2).

### The prognostic impact of the analysed genes

#### The correlation of gene expression data with distant metasasis free survival

**Figure 3:**
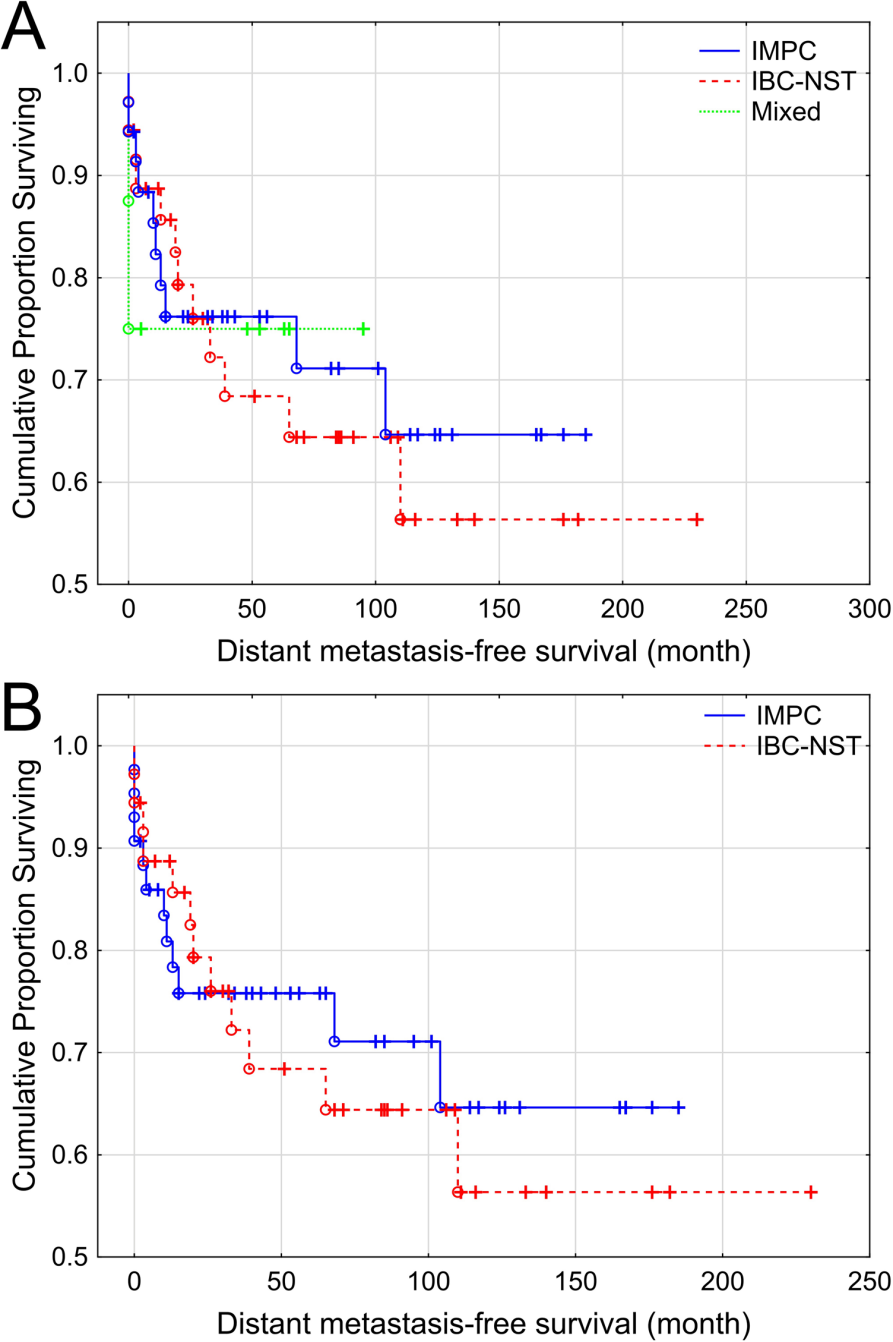
Correlation of histological tumor type with distant metastasis free survival. Based on distant metastasis-free survival, there was no significant difference between IMPC, IBC-NST and mixed IMPC/IBC-NST groups (p = 0.924) (**A**), as well as when comparing only pure IMPC and IBC-NST cases (p = 0.717) (**B**).

We compared DMFS intervals of the 36 pure IMPC and 36 IBC-NST patients as well as of the 8 mixed IMPC/IBC-NST patients. Statistical analysis revealed no significant differences in DMFS between IMPC, IBC-NST and mixed IMPC/IBC-NST patients (p = 0.924, Fig. 3A). Similar result was seen when comparing only pure IMPC and IBC-NST cases (p = 0.717, Fig. 3B).

**Figure 4:**
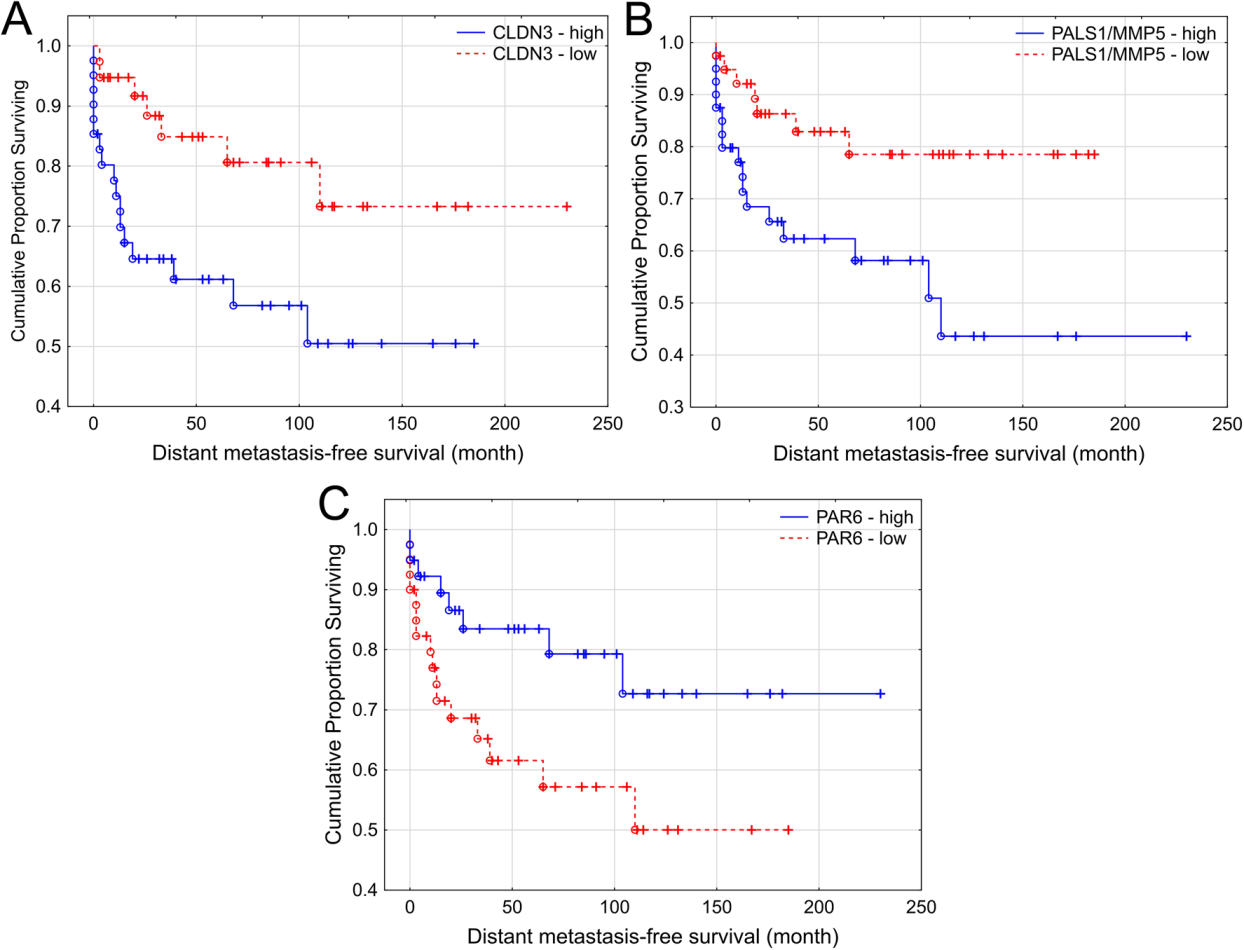
Correlation of gene expression data with distant metastasis free survival. High expression levels of CLDN3, PALS1 and low levels of PAR6 were associated with shorter DMFS intervals (p = 0.017, p = 0.013 and p = 0.045 respectively) (**A**–**C**).

DMFS intervals were compared with levels of mRNA expression of all examined genes in the entire cohort. High expression levels of CLDN3, PALS1 and low levels of PAR6 were associated with worse DMFS intervals (p = 0.017, p = 0.013 and p = 0.045 respectively) (Fig. 4A–C). No statistically significant association with DMFS was found in expression levels of other examined genes.

#### The correlation of gene expression with histological tumor grade.

**Figure 5:**
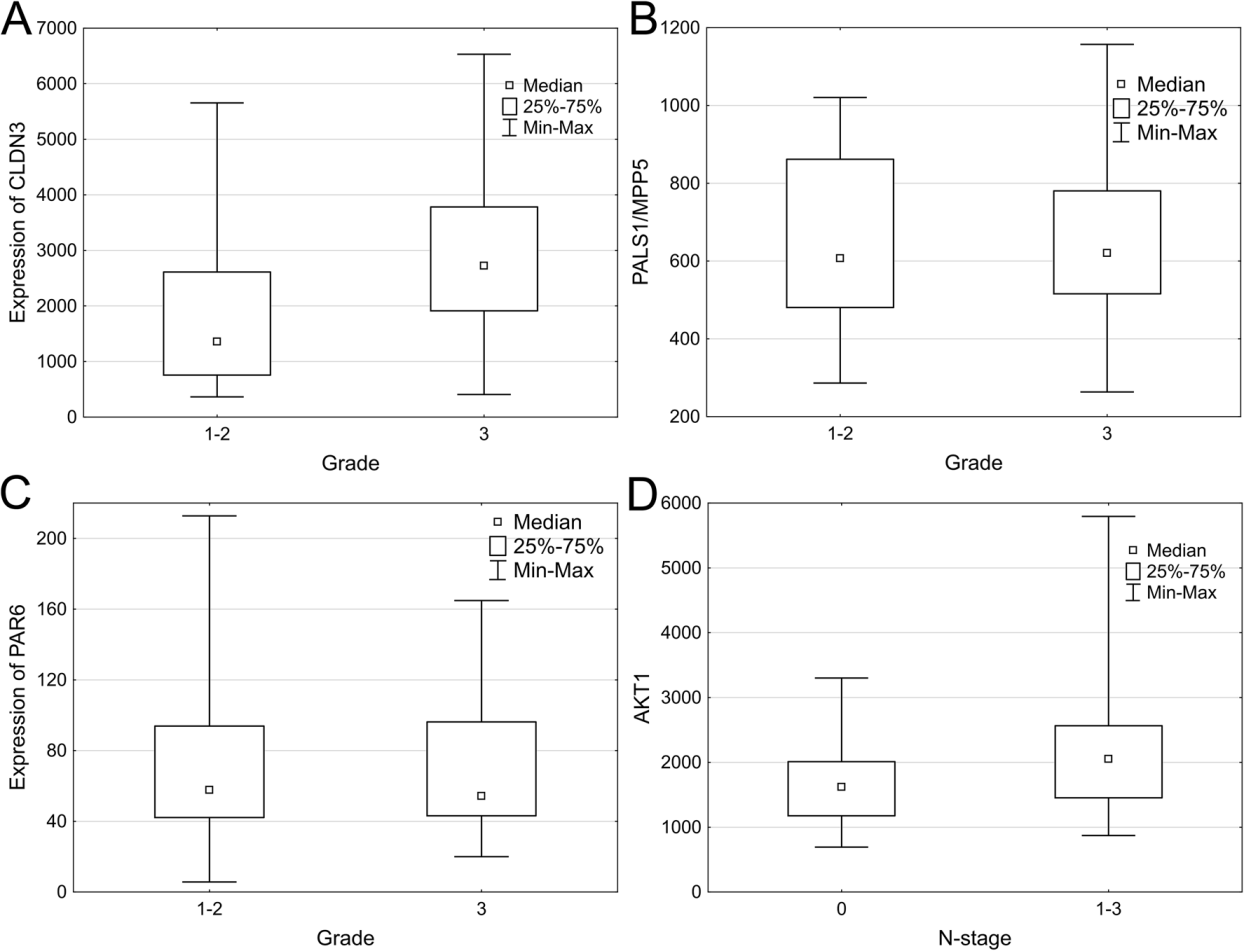
Association of gene expression with histological tumor grade and axillary lymph node metastasis. Expression levels of CLDN3 are associated with tumor grade (p = 0.0005) (**A**), while PALS1 and PAR6 expression are independent of tumor grade (p = 0.805 and p = 0.9 respectively) (**B**,**C**). High AKT1 expression levels (p = 0.033) (**D**) are associated with lymph node metastasis.

In the next step we analysed whether CLDN3, PALS1 and PAR6 expression levels were associated with tumor grade. CLDN3 showed association with tumor grade (grade 1 and 2 tumors were grouped together, while grade 3 tumors were in a separate group, Fig. 5A): higher CLDN3 levels were found in high grade tumors (p = 0.0005).

In univariate analysis, PALS1 and PAR6 expression levels showed no significant association with tumor grade, indicating that they might be grade independent prognostic factors (p = 0.805 and p = 0.9 respectively) (Fig. 5B,C). However, multivariate analysis confirmed only PALS1 as a grade independent prognostic factor (p = 0.007, Table 2).

**Table 2:** Multivariate analysis results.

	RR (95% CI)	p-value
Age	1.016 (0.974–1.06)	0.459
Grade (3 vs. 1–2)	4.96 (1.614–185.236)	0.005
Histology (IMPC vs. IBC-NST)	0.771 (0.332–1.793)	0.546
PAR6 (high vs. low)	0.508 (0.211–1.223)	0.131
PALS1 (high vs. low)	3.797 (1.44–10.01)	0.007

#### Gene expression data and axillary lymph node involvement

We have also examined whether the analysed gene expression levels have any correlation with lymph node status. We compared the expression levels between cases with pN0 status and lymph node positive cases. High expression levels of AKT1 was associated with lymph node metastasis (p = 0.033) (Fig. 5D). The examined chemokines and their receptors have not shown any association with lymph node involvement in our cohort.

#### Outcome analysis: comparison with KM Plotter database

**Figure 6:**
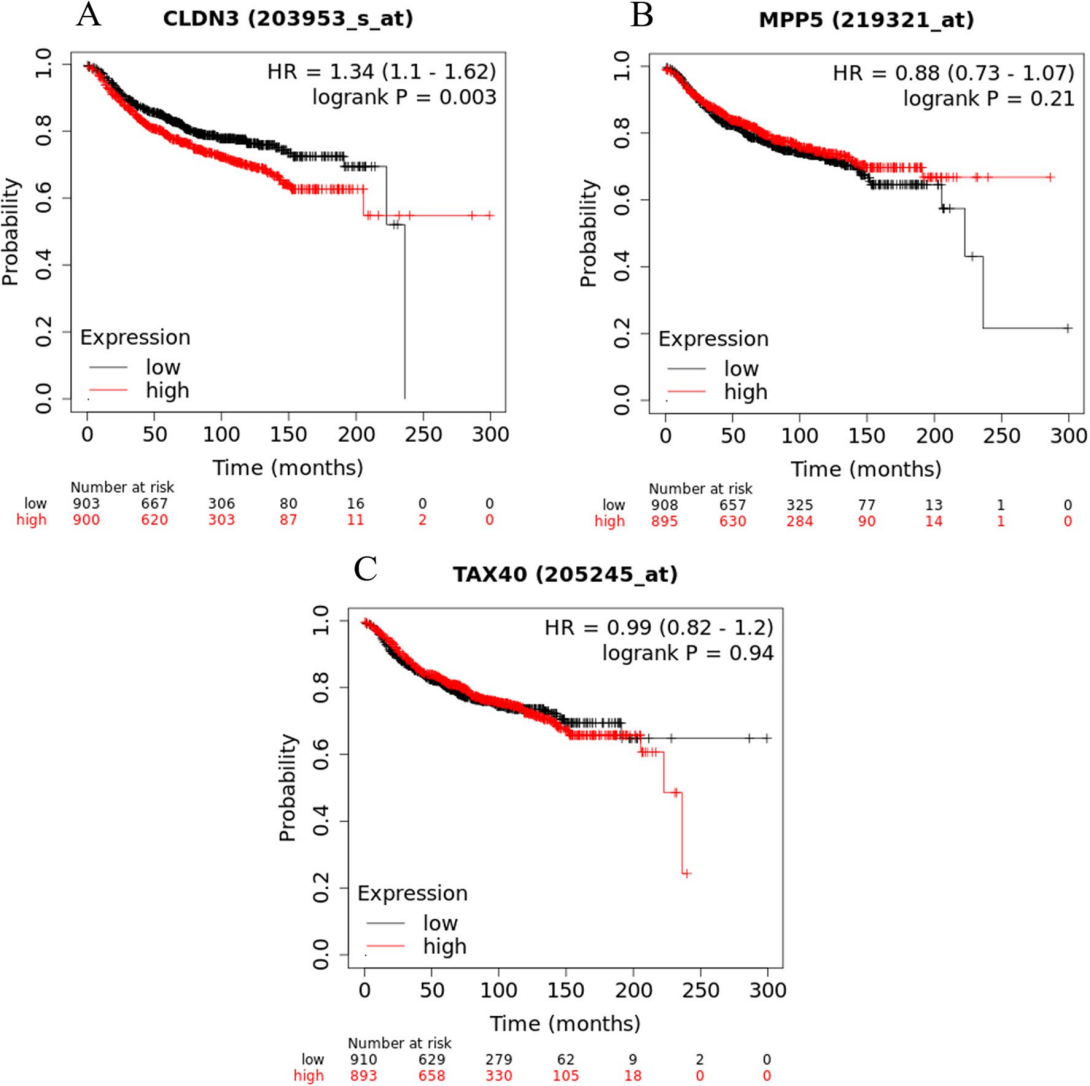
KM Plotter DMFS intervals in separate large cohort of breast carcinomas (regardless of histological type) from KM Plotter online database^40,41^. Correlation of high CLDN3 expression with shorter DMFS (**A**). No correlation with DMFS was found in case of PALS1 (MPP5) and PAR6 (TAX40) expression (**B**,**C**).

In our entire patient cohort high levels of CLDN3, PALS1 and low levels of PAR6 were associated with shorter DMFS. Similarly, according to the online KM Plotter database^40,41^, which provides data on their own, large cohort of breast carcinomas regardless of their histological type, high CLDN3 level is associated with shorter DMFS (p = 0.003), while in the KM Plotter database PALS1 and PAR6 showed no significant correlation with DMFS (Fig. 6).

## Discussion

Special and rare subtypes of breast cancer such as IMPC are in focus of current research, however, numerous questions still remain unanswered. Based on several earlier results, IMPCs are considered to have agressive clinical behavior, with high incidence of early lymph node metastasis, although according to recent studies overall survival rates are similar compared with IBC-NSTs^1,7,9,10^. The clusters of IMPC cells have a typical histological appearance with reversed polarity. The molecular mechanisms underlying the development of this structure are not fully understood. Some studies investigating cohorts of IMPCs with comparable case numbers to ours have shown that IMPCs are a heterogenous group of tumors with many genetic alterations different from IBC-NST^29,30,42,43^.

In our research study first we have examined the mRNA expression of carefully selected genes associated with cell adhesion and tight junction molecules as well as cell polarity complexes. Review of the literature revealed no other comprehensive studies examining such variety of related genes on the mRNA level. When comparing the mRNA expression levels in IMPC and IBC-NST, we have found 12 genes with significant differences in expression levels between the two groups. Among these were CLDNs -1, -3, -4, and -7, with CLDN1 showing significantly lower expression levels in IMPC, while the other three CLDNs presented higher expression levels in the IMPC group. Claudins, members of the tight junction complex are integral membrane proteins known to interact with signalling pathways^28^. Different patterns of claudin expression have been described in different molecular subtypes of breast cancer^32,33,44–51^.

The observations that high mRNA expression levels of CLDN3 are associated with high tumor grade in our study support that high CLDN3 expression is associated with poor prognosis of breast cancer. In our entire patient cohort high CLDN3 expression was associated with shorter DMFS.

It is known that Crumbs complex is one of the key members of apical polarity complexes which play crucial role in apico-basal polarity and directional cell migration. The complex is composed of several proteins including PALS (Proteins associated with LIN7) and PATJ (Pals1-associated tight junction) as its main components^25^. Disregulation of any of its components results in loss of cell polarity and tissue organization^31,52^. Gruel et al. found polarity protein LIN7A upregulated in IMPCs along with other polarity protein abnormal expression and localization^12^. In our study, although expression levels of PALS1 and PATJ were not significantly different in the IMPC and IBC-NST groups, we found significantly higher mRNA levels of LIN7A in the IMPC group, suggesting that LIN7A might be associated with the specific histological appearance. Nevertheless, our study highlighted that high mRNA levels of PALS1 were significantly associated with shorter DMFS and was found to be a grade independent prognostic factor, irrespective of histological subtype.

PAR protein complex, composed of PAR3/PAR6/aPKC proteins, also plays an important role in determining epithelial cell polarity^53^. PAR6 is often located at the apical part of the cells inducing asymmetric distribution of the cytoskeleton in epithelial cells. PAR6 has also been described as a signaling molecule in tumorigenesis and cancer development^54^. Nolan et al. have shown that PAR6 induces cell proliferation and is overexpressed in breast cancer^55^. Interestingly in our study low expression of PAR6 was significantly associated with worse DMFS in univariate analysis.

The Scribble polarity complex together with PAR-based and Crumbs-based complexes also participates in the regulation of epithelial cell polarity. It is composed of SCRIBBLE, LGL and DLG proteins, products of Drosophila tumor suppressor genes^56,57^. Studies have found that loss of DLG1 disintegrates tight junctions and is associated with poorly differentiated ductal breast carcinomas^58,59^. In line with these observations, our study has also shown lower mRNA expression levels of DLG1 in IMPC tumors.

Epithelial tumor cells undergo EMT to gain metastatic ability. EMT is activated by several transcription factor families (ZEB, SNAIL, TWIST etc.). These transcription factors act together with other intracellular signalling networks. It has been reported that other proteins such as TGF-β and the loss of E-cadherin also play roles in EMT^60,61^. Matsumura et al. have demonstrated that carcinoma-associated fibroblasts (CAFs) in tumor stroma drive the formation of tumor cell clusters composed of two distinct cancer cell populations: one highly epithelial state (showing high E-cadherin and low/negative ZEB1 expression), and one in a hybrid epithelial/mesenchymal state (showing low E-cadherin, high ZEB1 expression). These CAFs induce invasive and metastatic tumor cell clusters via epithelial–mesenchymal plasticity^62^. Interestingly in our study lower mRNA expression of ZEB1, SNAI2 and ITGA1 was found in the IMPC group, while CDH1 (E-cadherin) levels were significantly higher compared to IBC-NSTs. This finding suggest that IMPC tumor cell clusters seem to be in the highly epithelial state, and these tumor cells form clusters during stromal invasion and metastasis formation instead of using the traditional EMT pathway which is used by the IBC-NST tumors^63^.

The fact that IMPC tumors invade the stroma in form of clusters is underlined by our findings that significantly higher expression levels of OCLN are present in the IMPC group. OCLN is a member of the tight junction molecules containing the tetra-spanning MARVEL (MAL and related proteins for vesicle trafficking and membrane link) domain^64^. Our results may partly suggest that tumor cell clusters are stabilized during invasion with the help of this tight junction molecule.

AF6 (Afadin) has been described as an adherens junction protein. Tabariés et al. found that high protein levels of CLDN2 and AF6 in primary breast cancers were associated with poor survival^65^. Elloul et al. proved that phosphorylation of AF6 relocates the protein to the nucleus, and leads to increased breast cancer cell migration^66^. In our study AF6 also showed higher mRNA expression in the IMPC group, although regarding our overall findings, we could not establish any correlation between DMFS and AF6 levels.

Comparing our survival data with Kaplan Meier (KM) plotter we have also found that high CLDN3 expression is associated with worse DMFS. In our study PALS1 and PAR6 expression was also significantly associated with DMFS, although the KM Plotter database did not show the prognostic value of the above mentioned two genes.

Lymph node metastasis is commonly seen in patients with IMPC. The pathogenesis of the lymphotrophy of this cancer subtype is not fully understood. Several studies examined the role of certain chemokins and their receptors and other molecules in the development of lymph node metastasis in IMPCs on protein expression level and have found significant differences compared with IBC-NSTs^20,67,68^. In our study we could not demonstrate a significant difference in the mRNA expression levels of genes playing roles in chemotaxis (chemokines and their receptors) between the two groups. When comparing gene expression levels in all cases with and without lymph node metastasis, we found that high levels of AKT1 were associated with the presence of lymph node metastasis. AKT1 is a well known protein kinase that plays an important role in carcinogenesis by triggering tumor progression via the mammalian target of rapamycin (mTOR) signaling pathway^69^. AKT activation is also involved in the development of drug resistance in breast cancer and therefore is a potential target to overcome chemoresistance^70^.

Limitations of our study are our relatively low case numbers similarly to other molecular studies on IMPC tumors. We performed analysis of selected genes known to have key functions in cell adhesion, tight junction, cell polarity and major cancer signalling pathways rather than investigating several hundreds of genes. Most of our findings are in concordance with literature data, however we can not exclude that those genes not showing significant change or difference in expression levels in our cohort might present relevant alterations when investigated in a considerably larger group of patients.

## Conclusions

Among the 12 differentially expressed genes in IMPC, decreased CLDN1, DLG1 and increased LIN7A, CDH1 and OCLN expression may be associated with the unique histological appearance of this tumor type. In spite of the significant gene expression differences, DMFS was not markedly different between IMPC and IBC-NSTs in our cohort, which is in accordance with recent literature data. Nevertheless, high PALS1 and low PAR6 were associated with shorter DMFS and PALS1 proved to be a grade independent prognostic factor in the entire cohort. In both groups of tumors, the alterations of gene expression in the mTOR signaling pathway highlight the potential benefit of AKT/mTOR inhibitors in IMPCs, similarly to IBC-NSTs.

## Supplementary Information

Supplementary Figure S1.

Supplementary Table S1.

Supplementary Table S2.

Supplementary Table S3.

## Data Availability

The datasets used and analysed during the current study are available from the corresponding author on reasonable request.

## Abbreviations

IMPC: Invasive micropapillary carcinoma
EMA: Epithelial membrane antigen
IBC-NST: Invasive breast carcinoma-no special type
OS: Overall survival
DMFS: Distant metastasis free survival
ECM: Extracellular matrix
EMT: Epithelial-to-mesenchymal transition
TJ: Tight junction
TNF: Tumor necrosis factor
MARVEL: MAL and related proteins for vesicle trafficking and membrane link
mTOR: Mammalian target of rapamycin
SDF1: Stromal cell-derived factor-1
LZTS1: Leucine zipper putative tumor suppressor 1
