# Supplementary material for: Cell polarity and cell adhesion associated gene expression differences between invasive micropapillary and no special type breast carcinomas and their prognostic significance
Source: Sci Rep. 2021 Sep 16;11:18484. doi: 10.1038/s41598-021-97347-8 (PMC8446082; doi:10.1038/s41598-021-97347-8)
Supplement: Supplementary file 1 — Supplementary Figure S1. [file 41598_2021_97347_MOESM1_ESM.pdf]

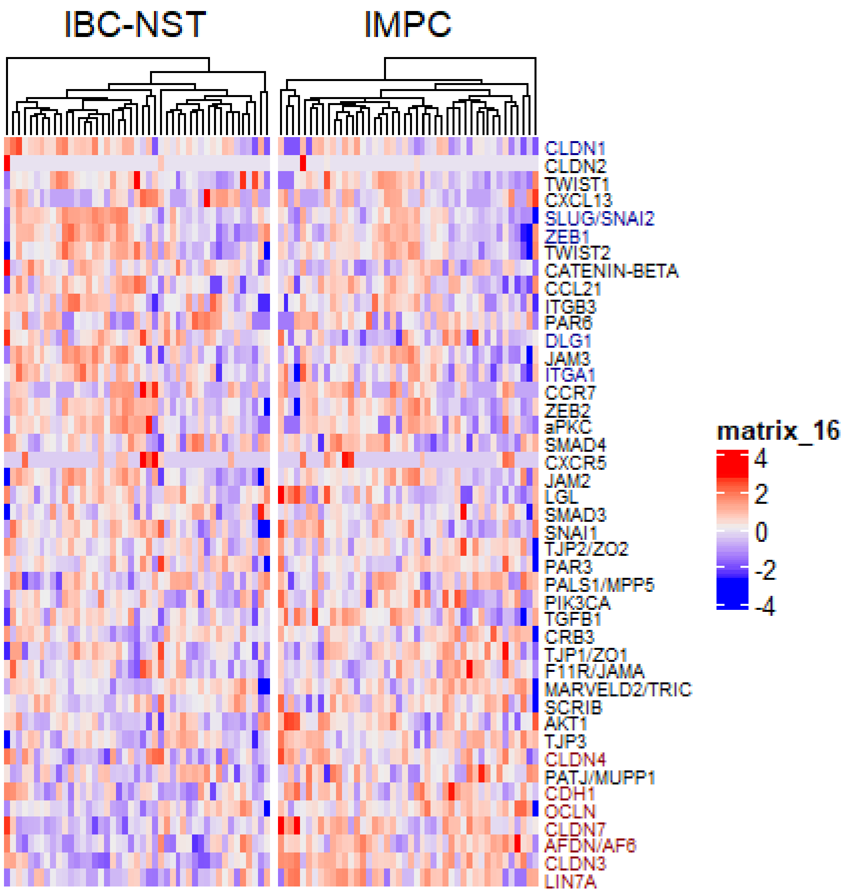

Supplementary Figure 1: Heatmap of gene expression levels in the IBC NST and IMPC group. Genes highlighted in blue showed significantly lower mRNA expression levels in the IMPC group, while those highlighted in red had higher expression levels compared to the NST group. The heatmap was generated with the ComplexHeatmap package (vs. 2.6.2) within R (vs. 4.0.4)[1].
https://www.bioconductor.org/packages/release/bioc/html/ComplexHeatmap.html

1. Gu, Z., Eils, R. & Schlesner, M. Complex heatmaps reveal patterns and correlations in multidimensional genomic data. *Bioinformatics* **32**, 2847-2849 (2016).
