# Supplementary material for: Cell polarity and cell adhesion associated gene expression differences between invasive micropapillary and no special type breast carcinomas and their prognostic significance
Source: Sci Rep. 2021 Sep 16;11:18484. doi: 10.1038/s41598-021-97347-8 (PMC8446082; doi:10.1038/s41598-021-97347-8)
Supplement: Supplementary file 2 — Supplementary Table S1. [file 41598_2021_97347_MOESM2_ESM.pdf]

Supplementary Table 1. Examined genes arranged in groups by their main function (these functions may overlap between groups).

	Gene name	Encoded protein
**Cell polarity genes**		
	CRB3	Crumbs Cell Polarity Complex Component 3
	PALS1/MPP5	Protein-Associated with Lin7
	PATJ/MUPP1	Pals1-Associated Tight Junction Protein
	PAR3	Partitioning-Defective Protein 3
	PAR6	Partitioning-Defective Protein 6
	aPKC	Atypical protein Kinase C
	SCRIB	Protein Scribble Homolog
	LGL	Drosophila Lethal Giant Larvae Protein Homolog-1
	DLG1	Discs Large MAGUK Scaffold Protein 1
	LIN7A	Lin7A Homolog Protein
**Tight junction and cell adhesion genes**		
	CLDN1	Claudin-1
	CLDN3	Claudin-3
	CLDN4	Claudin-4
	CLDN7	Claudin-7
	CLDN2	Claudin-2
	TJP1/ZO1	Tight Junction protein-1/Zonula occludens-1
	TJP2/ZO2	Tight Junction protein-2/Zonula occludens-2
	TJP3	Tight Junction protein-3
	MARVELD2/TRIC	Tricellulin
	F11R/JAMA	Junctional Adhesion Molecule-1
	JAM2	Junctional Adhesion Molecule-2
	JAM3	Junctional Adhesion Molecule-3
	CDH1	E-cadherin
	OCLN	Occludin
	ITGA1	Interferon-alpha-1
	ITGB3	Interferon-beta-3
**Genes in cancer signaling pathways**		
	SNAI1	Snail Family Transcriptional Repressor 1
	SLUG/SNAI2	Snail Family Transcriptional Repressor 2
	ZEB1	Zinc Finger E-Box Binding Homeobox 1
	ZEB2	Zinc Finger E-Box Binding Homeobox 2
	SMAD3	SMAD Family Member Related Protein 3
	SMAD4	SMAD Family Member Related Protein 4
	TGFB1	Tumor Growth Factor Beta 1
	TWIST1	Twist Related Protein 1

	TWIST2	Twist Related Protein 2
	CATENIN-BETA	Catenin-beta
	AFDN/AF6	Afadin
	PIK3CA	Phosphatidylinositol-4,5-Bisphosphate 3Kinase Catalytic Subunit Alpha
	AKT1	AKT Serine/Threonine Kinase 1
**Chemokines and their receptors**		
	CCR7	C-C Motif Chemokine Receptor 7
	CCL21	C-C Motif Chemokine Ligand 21
	CXCR5	C-X-C Motif Chemokine Receptor 5
	CXCL13	C-X-C Motif Chemokine Ligand 13
**Housekeeping genes**		
	AMMECR1L	AMMECR1L-like protein
	CC2D1B	Coiled-Coil And C2 Domain Containing 1B
	SAP130	Sin3A Associated Protein 130
	ZNF143	Zinc Finger Protein 143
	NUBP1	Nucleotide Binding Protein 1
