# Supplementary material for: Cell polarity and cell adhesion associated gene expression differences between invasive micropapillary and no special type breast carcinomas and their prognostic significance
Source: Sci Rep. 2021 Sep 16;11:18484. doi: 10.1038/s41598-021-97347-8 (PMC8446082; doi:10.1038/s41598-021-97347-8)
Supplement: Supplementary file 3 — Supplementary Table S2. [file 41598_2021_97347_MOESM3_ESM.pdf]

Supplementary Table 2. Detailed NanoString Design Report

	Customer Identifier	Accession	Position	Target Sequence	Tm CP	Tm RP	HUGO Gene	NSID
1	AFDN/AF6	NM_001040000.2	3781-3880	GCCTAGACCTGAAGCCTACCCCATCCCCACTCAGACGTACACCAGAGAGTATTTTACCTTCCCAGCTTCCAAATCCCAGGATCGGATGGCTCCTCCTCAG	85	82	AFDN	NM_001040000.2:3780
2	AKT1	NM_001014432.1	1276-1375	GGACGGGCACATTAAGATCACAGACTTCGGGCTGTGCAAGGAGGGGATCAAGGACGGTGCCACCATGAAGACCTTTTGCGGCACACCTGAGTACCTGGCC	85	84	AKT1	NM_001014432.1:1275
3	AMMECR1L	NM_031445.2	253-352	TGGGAAAAAGACGTTGTGTTCCTCCACTCGAGCCCAAGTTGGCAGCAGGCTGTTGTGGGGTCAAGAAGCCCAAATTATCTGGAAGTGGAACGCACAGTCA	83	83	AMMECR1L	NM_031445.2:252
4	CC2D1B	NM_032449.2	1387-1486	GATGCATGAGCGCATTGCCAAGCAATATCAAGATGCTATTCGAGCACACCGAGCAGGACGGAAAGTCAACTTTGCTGAATTGCCTGTTCCTCCAGGATTT	82	82	CC2D1B	NM_032449.2:1386
5	CCL21	NM_002989.2	181-280	CTCAAGTACAGCCAAAGGAAGATTCCCGCCAAGGTTGTCCGCAGCTACCGGAAGCAGGAACCAAGCTTAGGCTGCTCCATCCCAGCTATCCTGTTCTTGC	81	82	CCL21	NM_002989.2:180
6	CCR7	NM_001838.2	1611-1710	TTCCGAAAACCAGGCCTTATCTCCAAGACCAGAGATAGTGGGGAGACTTCTTGGCTTGGTGAGGAAAAGCGGACATCAGCTGGTCAAACAAACTCTCTGA	82	79	CCR7	NM_001838.2:1610
7	CDH1	NM_004360.2	536-635	GAATTGCTCACATTTCCCAACTCCTCTCCTGGCCTCAGAAGACAGAAGAGAGACTGGGTTATTCCTCCCATCAGCTGCCCAGAAAATGAAAAAGGCCCAT	80	78	CDH1	NM_004360.2:535
8	CLDN1	NM_021101.3	411-510	GCAAAGTCTTTGACTCCTTGCTGAATCTGAGCAGCACATTGCAAGCAACCCGTGCCTTGATGGTGGTTGGCATCCTCCTGGGAGTGATAGCAATCTTTGT	83	82	CLDN1	NM_021101.3:410
9	CLDN2	NM_020384.3	2541-2640	GGCTTCTGAAAAATTCCAGCACACCTCCTCGAACCTCATTGTCAGCAGAGAGGGCCCATCTGTTGTCTGTAACATGCCTTTCACATGTCCACCTTCTTGC	84	81	CLDN2	NM_020384.3:2540
10	CLDN3	NM_001306.3	607-706	TGCTCACCCTCGTGCCGGTGTCCTGGTCGGCCAACACCATTATCCGGGACTTCTACAACCCCGTGGTGCCCGAGGCGCAGAAGCGCGAGATGGGCGCGGG	82	83	CLDN3	NM_001306.3:606
11	CLDN4	NM_001305.3	1243-1342	GGGAGCTGGCTTCTGCTGGCCAGGATAGCTTAACCCTGACTTTGGGATCTGCCTGCATCGGCGTTGGCCACTGTCCCCATTTACATTTTCCCCACTCTGT	86	81	CLDN4	NM_001305.3:1242
12	CLDN7	NM_001307.3	176-275	AATTTTGTCTGGAACCACCCTCCCGGCGTATCCTACTCCCTGTGCCGCGAGGCCATCGCTTCACTGGAGGGGTCGATTTGTGTGTAGTTTGGTGACAAGA	83	81	CLDN7	NM_001307.3:175
13	CRB3	NM_139161.3	301-400	GAATAGCACTGTTTTGCCTTCATCCACCAGCTCCAGCTCCGATGGCAACCTGCGTCCAGAAGCCATCACTGCTATCATCGTGGTCTTCTCCCTCTTGGCT	83	84	CRB3	NM_139161.3:300
14	CATENIN-BETA	NM_001098210.1	1816-1915	TCTTGCCCTTTGTCCCGCAAATCATGCACCTTTGCGTGAGCAGGGTGCCATTCCACGACTAGTTCAGTTGCTTGTTCGTGCACATCAGGATACCCAGCGC	82	81	CTNNB1	NM_001098210.1:1815
15	CXCL13	NM_006419.2	211-310	AGACGCTTCATTGATCGAATTCAAATCTTGCCCCGTGGGAATGGTTGTCCAAGAAAAGAAATCATAGTCTGGAAGAAGAACAAGTCAATTGTGTGTGTGG	79	80	CXCL13	NM_006419.2:210
16	CXCR5	NM_001716.3	2619-2718	ACGTCCCTTTTTTCTCTGAGTATCTCCTCGCAAGCTGGGTAATCGATGGGGGAGTCTGAAGCAGATGCAAAGAGGCAAGAGGCTGGATTTTGAATTTTCT	79	82	CXCR5	NM_001716.3:2618
17	DLG1	NM_001098424.1	1461-1560	CTCAGCCTGTTGATAACCATGTTAGCCCATCTTCCTTCTTGGGCCAGACACCAGCATCTCCAGCCAGATACTCCCCAGTTTCTAAAGCAGTACTTGGAGA	80	82	DLG1	NM_001098424.1:1460
18	F11R/JAMA	NM_144503.1	3651-3750	GGAAGCAGAGGTGATTCATGGCTCTGTGAATTTGAGGTGAATGGTTCCTTATTGTCTAGGCCACTTGTGAAGAATATGAGTCAGTTATTGCCAGCCTTGG	81	79	F11R	NM_144503.1:3650
19	ITGA1	NM_181501.1	1876-1975	AAGTGGCAAGACTATAAGGAAAGAGTATGCACAACGTATTCCATCAGGTGGGGATGGTAAGACACTGAAATTTTTTGGCCAGTCTATCCACGGAGAAATG	83	81	ITGA1	NM_181501.1:1875
20	ITGB3	NM_000212.2	4486-4585	GAATAAGCCTTGGAATTAGATATGGGGCAATGACTGAGCCCTGTCTCACCCATGGATTACTCCTTACTGTAGGGAATGGCAGTATGGTAGAGGGATAAAT	82	79	ITGB3	NM_000212.2:4485
21	JAM2	NM_001270407.1	841-940	CCATCATGTGAAGTACCCTCTTCTGCTCTGAGTGGAACTGTGGTAGAGCTACGATGTCAAGACAAAGAAGGGAATCCAGCTCCTGAATACACATGGTTTA	83	83	JAM2	NM_001270407.1:840
22	JAM3	NM_032801.3	891-990	AATTATTGGGGGGGTTCTGGTTGTCCTTGCTGTACTGGCCCTGATCACGTTGGGCATCTGCTGTGCATACAGACGTGGCTACTTCATCAACAATAAACAG	83	83	JAM3	NM_032801.3:890
23	LIN7A	NM_004664.2	1028-1127	TAAATTCTTTACCAAGGCAACTCAACACCTTCTTTCTCTGGGCTTGAACCGCCACTGCTCACGTGGGCTTTACATACATTGACCTTCCATTCACTGCAGT	78	82	LIN7A	NM_004664.2:1027
24	LGL	NM_004140.3	1035-1134	TGCTTCGAGCCGAGACATTGGTGACGCTGGACTTCACTTCCCGCATCATCGACTTCTTCACAGTGCACAGCACACGGCCCGAGGATGAATTTGATGACCC	83	83	LLGL1	NM_004140.3:1034
25	MARVELD2/TRIC	NM_001038603.1	666-765	TTGGGGGCCGGTGTCTTTGCTTGTGTCACAGCTTACATTCACAAGGACAGTGAGTGGTACAACTTGTTTGGATATTCACAACCGTATGGCATGGGAGGCG	82	80	MARVELD2	NM_001038603.1:665
26	PALS1/MPP5	NM_022474.2	1111-1210	ACATATTCAGGCACTTTTACTGGCCCACGATAAGGTTGCTGAGCAGGAAATGCAGCTAGAGCCCATTACAGATGAGAGAGTTTATGAAAGTATTGGCCAG	83	83	MPP5	NM_022474.2:1110
27	NUBP1	NM_002484.3	231-330	GACTGTAAAACACAAAATCTTGGTATTGTCTGGGAAAGGCGGTGTTGGGAAAAGCACATTCAGCGCCCACCTTGCCCATGGCCTAGCAGAGGATGAAAAC	83	84	NUBP1	NM_002484.3:230
28	OCLN	NM_002538.3	1976-2075	GTTGGAGACTATGATAGACAGAAAACATAGAAGGCTGATGCCAAGTTGTTTGAGAAATTAAGTATCTGACATCTCTGCAATCTTCTCAGAAGGCAAATGA	82	78	OCLN	NM_002538.3:1975
29	PAR3	NM_019619.2	1061-1160	GGTTCCATGTGGTTCCTGCAGCAAATAAAGAGCAGTATGAACAACTATCCCAAAGTGAGAAGAACAATTACTATTCAAGCCGTTTTAGCCCTGACAGCCA	82	79	PARD3	NM_019619.2:1060
30	PAR6	NM_016948.2	144-243	TGAAGAGCAAATTTGACGCCGAGTTCCGACGCTTCGCGCTGCCTCGCGCTTCGGTGAGCGGCTTCCAGGAGTTCTCGCGGTTGCTGCGGGCGGTGCACCA	83	87	PARD6A	NM_016948.2:143
31	PATJ/MUPP1	NM_176877.2	1395-1494	GGGTTTTGCCAACCATGATGTTGTTGAAGTATTACGAAATGCAGGGCAGGTGGTACACCTAACCCTAGTTCGAAGGAAGACATCCTCATCTACTTCTCCA	80	83	PATJ	NM_176877.2:1394
32	PIK3CA	NM_006218.2	2446-2545	CCTCAGGCTTGAAGAGTGTCGAATTATGTCCTCTGCAAAAAGGCCACTGTGGTTGAATTGGGAGAACCCAGACATCATGTCAGAGTTACTGTTTCAGAAC	76	77	PIK3CA	NM_006218.2:2445
33	aPKC	NM_002737.2	681-780	AAAACCATCCGCTCCACACTAAATCCGCAGTGGAATGAGTCCTTTACATTCAAATTGAAACCTTCAGACAAAGACCGACGACTGTCTGTAGAAATCTGGG	81	83	PRKCA	NM_002737.2:680
34	SAP130	NM_024545.3	3091-3190	GATCTCCACCGAATAAACGAACTGATACAGGGAAATATGCAGAGGTGTAAACTTGTGATGGATCAAATCAGTGAAGCCAGAGACTCCATGCTTAAGGTTT	83	81	SAP130	NM_024545.3:3090
35	SCRIB	NM_182706.3	5018-5117	AGCTCCAGCACCACCCTTGCCCCAAGTCTTTTAACCTGGGTGTTAGCATTTTAAAGAGACCCCACAGGAGTTCTGGCCTGTGACTAACTAACTGCCCCAC	84	84	SCRIB	NM_182706.3:5017
36	SMAD3	NM_005902.3	4221-4320	TTAAAGGACAGTTGAAAAGGGCAAGAGGAAACCAGGGCAGTTCTAGAGGAGTGCTGGTGACTGGATAGCAGTTTTAAGTGGCGTTCACCTAGTCAACACG	79	80	SMAD3	NM_005902.3:4220
37	SMAD4	NM_005359.3	1371-1470	AGGTTGCACATAGGCAAAGGTGTGCAGTTGGAATGTAAAGGTGAAGGTGATGTTTGGGTCAGGTGCCTTAGTGACCACGCGGTCTTTGTACAGAGTTACT	79	80	SMAD4	NM_005359.3:1370
38	SNAI1	NM_005985.2	64-163	GACCACTATGCCGCGCTCTTTCCTCGTCAGGAAGCCCTCCGACCCCAATCGGAAGCCTAACTACAGCGAGCTGCAGGACTCTAATCCAGAGTTTACCTTC	85	86	SNAI1	NM_005985.2:63
39	SLUG/SNAI2	NM_003068.3	741-840	GCGTTTTCCAGACCCTGGTTGCTTCAAGGACACATTAGAACTCACACGGGGGAGAAGCCTTTTTCTTGCCCTCACTGCAACAGAGCATTTGCAGACAGGT	80	81	SNAI2	NM_003068.3:740
40	TGFB1	NM_000660.3	1261-1360	TATATGTTCTTCAACACATCAGAGCTCCGAGAAGCGGTACCTGAACCCGTGTTGCTCTCCCGGGCAGAGCTGCGTCTGCTGAGGCTCAAGTTAAAAGTGG	79	82	TGFB1	NM_000660.3:1260
41	TJP1/ZO1	NM_003257.3	6278-6377	CACATTTTTCTTAGGGAAGGATACAAAAGCATGTGAGACTGGTTCCATGGCCTCTTCAGATCTCTAACTTCACCATATTACCACAGACATACTAACCAGC	78	80	TJP1	NM_003257.3:6277
42	TJP2/ZO2	NM_004817.2	2786-2885	AGCCAATGATAGCTGGTTTGGCAGCTTAAAGGACACTATTCAGCATCAGCAAGGAGAAGCGGTTTGGGTCTCTGAAGGAAAGATGGAAGGGATGGATGAT	81	82	TJP2	NM_004817.2:2785
43	TJP3	NM_014428.1	1926-2025	AGTGGTGTTGCGAGAAGCCAGTTTCAAGCGCCCGGTAGTGATCCTGGGACCCGTGGCCGACATTGCTATGCAGAAGTTGACTGCTGAGATGCCTGACCAG	86	86	TJP3	NM_014428.1:1925
44	TWIST1	NM_000474.3	36-135	CAACTCCCAGACACCTCGCGGGCTCTGCAGCACCGGCACCGTTTCCAGGAGGCCTGGCGGGGTGTGCGTCCAGCCGTTGGGCGCTTTCTTTTTGGACCTC	87	82	TWIST1	NM_000474.3:35
45	TWIST2	NM_057179.2	483-582	AGCTGAGCAAGATCCAGACGCTCAAGCTGGCCGCCAGGTACATAGACTTCCTCTACCAGGTCCTGCAGAGCGACGAGATGGACAATAAGATGACCAGCTG	85	85	TWIST2	NM_057179.2:482
46	ZEB1	NM_001128128.1	1451-1550	TTACAAAATGGGGTTTTCACTGGTGGTGGCCCATTACAGGCAACCAGTTCTCCTCAGGGCATGGTGCAAGCTGTTGTTCTGCCAACAGTTGGTTTGGTGT	82	83	ZEB1	NM_001128128.1:1450
47	ZEB2	NM_001171653.1	241-340	GTTTGTGTTTCCTGGAGTGGCCGAAAGAGATCAGTTCTAACCTGCTCTGCAGGAATAACGGTCCTGCCTCCCGACACTCTTGGCGAGGTTTTTGTACAGT	81	83	ZEB2	NM_001171653.1:240
48	ZNF143	NM_003442.5	926-1025	TGAGCATGCAGGCTGTGGGAAGGCATTTGCAACAGGTTATGGATTAAAAAGTCACGTCAGAACTCATACAGGAGAAAAGCCATATCGGTGTTCGGAAGAT	84	83	ZNF143	NM_003442.5:925
