# Supplementary material for: Cell polarity and cell adhesion associated gene expression differences between invasive micropapillary and no special type breast carcinomas and their prognostic significance
Source: Sci Rep. 2021 Sep 16;11:18484. doi: 10.1038/s41598-021-97347-8 (PMC8446082; doi:10.1038/s41598-021-97347-8)
Supplement: Supplementary file 4 — Supplementary Table S3. [file 41598_2021_97347_MOESM4_ESM.pdf]

Supplementary Table 3.

Difference of gene expression pattern compared between IMPC and IBC-NST groups.

Gene name	p-value
AFDN/AF6	0.000005
AKT1	0.120617
CATENIN-BETA	0.091492
CCL21	0.507676
CCR7	0.520147
CDH1	0.01176
CLDN1	0.004673
CLDN2	0.489268
CLDN3	0.000005
CLDN4	0.002431
CLDN7	0.000131
CRB3	0.186806
CXCL13	0.949877
CXCR5	0.839064
DLG1	0.002207
F11R/JAMA	0.17422
ITGA1	0.044779
ITGB3	0.162277
JAM2	0.666935
JAM3	0.236139
LGL	0.054898
LIN7A	0.000081
MARVELD2/TRIC	0.054898
OCLN	0.000233
PALS1/MPP5	0.477198
PAR3	0.80521
PAR6	0.552001
PATJ/MUPP1	0.074377
PIK3CA	0.30303
SCRIB	0.221187
SLUG/SNAI2	0.007495
SMAD3	0.919118
SMAD4	0.471225
SNAI1	0.489268
TGFB1	0.483212
TJP1/ZO1	0.053687

TJP2/ZO2	0.632143
TJP3	0.054898
TWIST1	0.340798
TWIST2	0.236139
ZEB1	0.049064
ZEB2	0.376214
aPKC	0.760644
